# Mesenchymal stem cells: potential application for the treatment of hepatic cirrhosis

**DOI:** 10.1186/s13287-018-0814-4

**Published:** 2018-03-09

**Authors:** Yongting Zhang, Yuwen Li, Lili Zhang, Jun Li, Chuanlong Zhu

**Affiliations:** 1 Department of Infectious Disease, the First Affiliated Hospital with Nanjing Medical University, Nanjing, China; 2 Department of Pediatrics, the First Affiliated Hospital with Nanjing Medical University, Nanjing, China

**Keywords:** Mesenchymal stem cells, Cirrhosis, Homing

## Abstract

Nowadays, orthotopic liver transplantation is considered the most efficient approach to the end stage of chronic hepatic cirrhosis. Because of the limitations of orthotopic liver transplantation, stem cells are an attractive therapeutic option. Mesenchymal stem cells (MSCs) especially show promise as an alternative treatment for hepatic cirrhosis in animal models and during clinical trials. Nevertheless, the homing of transplanted MSCs to the liver occurs in limited numbers. Therefore, we review the strategies for enhancing the homing of MSCs, mainly via the delivery routes, optimizing cell culture conditions, stimulating the target sites, and genetic modification.

## Background

Cirrhosis is the end stage of progressive fibrosis that is caused by various reasons and that responds poorly to medical conservative treatment. Chronic damage to the liver leads to the extensive accumulation of extracellular matrix (ECM) among the hepatocytes. Epidemiological data state that 1.03 million cirrhotic patients worldwide die each year from severe associated complications [1].

Currently, liver transplantation is the most effective therapy for advanced hepatic diseases. Among those fortunate enough to receive liver transplantation, the survival rates at 3, 12, and 36 months are 94%, 88%, and 79%, respectively [2]. However, we should be take into account the lack of donor organs, the high costs, and the long-term use of immunosuppressants after transplantation. Thus, there is an urgent need to find alternative therapeutic strategies. Recent studies have shown that hepatocytes in the cirrhotic liver still have the potential to regenerate, but there is an imbalance between regeneration and necrosis [3]. A potential hypothesis states that a fully functioning part of the liver could be created through the proliferation of the infused cells that will remodel the injured liver. It is doubtful whether increasing the number of hepatocytes alone would be an effective treatment for the patients.

Based on the proof-of-concept, hepatocytes were transplanted to treat liver-related diseases [4]. Because of the limited number of hepatocytes and the lack of their proliferation and stability in vitro, the efficacy of grafted hepatocytes decreased progressively. Hence, it is crucial to find another readily available cell source.

This review aims to highlight all currently available evidence regarding the use of stem cells for treatment of liver cirrhosis and to determine whether there is any factual basis for their potential.

## Stem cells in regenerative medicine

**Fig. 1:**
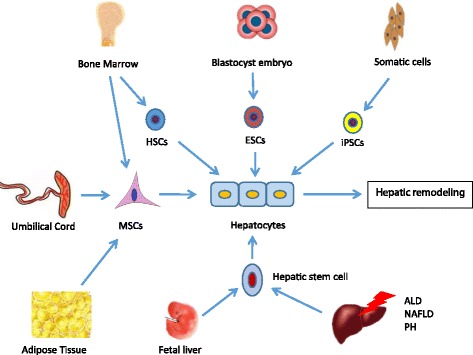
The different types of stem cells isolated from different tissues differentiate into hepatocytes. ALD alcoholic liver disease, ESCs embryonic stem cells, HSCs hematopoietic stem cells, iPSCs induced pluripotent stem cells, MSCs mesenchymal stem cells, NAFLD nonalcoholic fatty liver disease, PH partial hepatectomy

Stem cells, termed as clonogenic undifferentiated cells, cannot just self-renew indefinitely but can differentiate into a variety of cell lineages, including pluripotent embryonic stem cells (ESCs), induced pluripotent stem cells (iPSCs), hematopoietic stem cells (HSCs), hepatic stem cells, mesenchymal stem cells (MSCs), and so forth (Fig. 1).

Splenic teratomas could be formed after infusion of ESCs [5]. The application of ESCs is therefore limited because of their potential for malignancy. iPSCs are artificially derived from a nonpluripotent cell and thus ethical issues remain the major obstacle to their clinical administration. Furthermore, the only available source of HSCs is the hematopoietic system, and this restricts their clinical application. Hepatic stem cells have been identified in fetal as well as mature liver. During embryonic development, the cells within the liver bud are recognized as hepatoblasts which are bipotent, giving rise to both hepatocytes and bile-duct epithelial cells. Moreover, cells in the ductal plates in fetal and neonatal livers are also hepatic stem cells. Their capacity to repopulate the liver upon transplantation is also well studied in animal models [6, 7]. Hepatic progenitor cells (HPCs), also defined as hepatic stem cells, are rare in normal adult livers (0.01%), located in the Canals of Hering, and all regenerative responses are mainly granted by mature hepatocytes except in certain disease states [7].

HPCs are activated after liver injury, such as alcoholic liver disease (ALD) and nonalcoholic fatty liver disease (NAFLD) [8]. Oxidative stress, which plays the main role in the pathogenesis of ALD and NAFLD, promotes the accumulation and differentiation of HPCs into hepatocytes [9, 10]; furthermore, HPCs can differentiate into hepatocytes in vivo and promote liver regeneration after partial hepatectomy or acute toxic liver injury [11]. This suggests that infusion of the progenitor cells may alleviate the damage of hepatocytes which is caused by long-lasting oxidative stress or partial hepatectomy.

The proliferation of HPCs as a response to chronic liver damage is minimal [11] and is correlated with the severity and localization of the inflammatory infiltrate [12]. Manipulation of the HPC microenvironment may be used as a therapeutic approach for the alleviation of liver insufficiency [11, 12].

In addition, evidence has suggested that mesenchymal cells through the processes of mesenchymal-epithelial or epithelial-mesenchymal transition (MET/EMT) may contribute to adult liver regeneration during chronic liver injury [13]. Mesenchymal cells in the liver may be derived not only from their own progenitor cells but also from the bone marrow (BM) by migrating to the injured liver [14, 15], although this statement is controversial. This suggests that not only HPCs but also mesenchymal cells simultaneously contribute to the initiation and development of liver diseases, although the mechanisms remain unclear [16]. This indicates that the interaction between HPCs and mesenchymal cells is important for remodeling of injured liver. The accumulating evidence suggests that HPCs could be the best alternative treatment for hepatic damage; however, HPCs may cause carcinogenesis and fibrogenesis, as has been shown in vitro [6]. Before thorough viewing of their therapeutic potential, a better knowledge of the factors that determine HPC differentiation and their possible malignant transformation is necessary.

The therapeutic potential of MSCs has been extensively investigated as well as their differentiation, immunoregulatory properties, and secretion of trophic factors. In contrast to ESCs, iPSCs, and HPCs, MSCs do not have any ethical problems and have become the ideal alternative.

During the past few years, MSCs have been mainly isolated from the bone marrow (BM-MSCs). Alternative sources of MSCs have been proposed, such as from adipose tissue (AD-MSCs), umbilical cord blood (CB-MSCs), umbilical cord (UC-MSCs), and amniotic fluid.

## The application of MSCs

BM-MSCs are capable of undergoing differentiation into hepatic cells and recovering liver function, indicated by the apoptosis of hepatic stellate cells, decreased transforming growth factor (TGF)-β1, and alpha-smooth muscle actin (α-SMA) gene expression [17]. AD-MSCs, which are more immunocompatible and easier to isolate than BM-MSCs, have a protective role against liver fibrosis [18]. UC-MSCs show a more beneficial immunogenic profile and stronger overall immunosuppressive potential than BM-MSCs [19].

Although MSC differentiation into hepatocytes has been demonstrated in vivo, evidence suggests that various trophic and immunomodulatory factors play a key therapeutic role in the treatment of liver fibrosis. The trophic factors, which are secreted by MSCs, prevent apoptosis of hepatocytes with the help of antiapoptotic factors (hepatocyte growth factor (HGF) and insulin-like growth factor (IGF-1)), angiogenetic factors (vascular endothelial growth factor (VEGF)), mitogenetic factors (epidermal growth factor (EGF), HGF, and nerve growth factor (NGF)), and TGF-α [20, 21]. Because of the smaller and less complex immunogenic potency, MSC-free therapy might constitute a better alternative treatment.

Further clinical trials have evaluated the efficiency of transplanted MSCs for treating patients with liver fibrosis. Several clinical trials have been designed to evaluate their therapeutic potential in hepatic cirrhosis treatment [22–26] (Table 1). The results of the studies seem to be promising, with improvements in model for end-stage liver disease (MELD) score and metabolic parameters, but data on histological improvement are weak. Long-term outcomes after UC-MSC treatment would be preferable for patients with liver cirrhosis [22, 23], although the short-term efficacy of infused BM-MSCs is favorable [24–26]. It should be noted that the number of infused cells, the delivery route, and the frequency of injection per patient vary in the studies. Different sources of MSCs and various populations of patients may be more convincing for any therapeutic effect. Moreover, AD-MSCs and UC-MSCs have better immunocompatibility, and they are more vitalized and much easier to isolate than BM-MSCs from older patients [18, 19]. The efficacy of autologous BM-MSCs may suffer from aging differentiation and deficiency in vitality [18, 22]. In contrast, allogeneic UC-MSCs are free from these limitations [19, 22]. Furthermore, for prognosis and better analysis on the difference between stem cells, the follow-up time of patients should be prolonged with the creation of time points. The results are also limited because of small sample sizes and absence of control groups [22–26]. Currently, there are no standardized protocols for clinical trials and it is not possible to monitor whether the infused MSCs home to the targeted organs or not.

**Table 1:** MSCs in clinical trials treating liver fibrosis

Cell source	Delivery route	No. of cells	Patient population	No. of patients	Follow-up period	Efficacy	Limitations	Reference
UC-MSCs	Intravenous	5 × 10^5^/kg, three times	Chronic hepatitis B	30 treatment, 15 control	12 months	Improvement of liver function and MELD score; reduced ascites	No track of the infused UC-MSCs and the histological evidence in the studied patients	[22]
UC-MSCs	Intravenous	5 × 10^5^/kg, three times	Primary biliary cirrhosis	7 treatment	48 weeks	Decrease in serum ALP and γ-GGT; alleviation of fatigue and pruritus	No track of the infused UC-MSCs and histological evidence alterations in the studied patients; less detailed follow-up time points	[23]
BM-MSCs	Intravenous infusion	1 × 10^7^/kg	Liver cirrhosis due to hepatitis C virus	15 treatment,10 control	6 months	Improvement in the frequency of encephalopathy, jaundice, ascites, bleeding tendency, and lower limb edema	Less detailed follow-up time points	[24]
Autologous BM-MSCs	Hepatic artery	0.75 ± 0.50× 10^6^/patient	Hepatitis B virus cirrhosis	27 treatment,29 control	24 weeks	Significant improvement in liver function	During follow-up, patients were lost about 1/3	[25]
Autologous BM-MSCs	Peripheral vein	1 × 10^6^/kg	End-stage liver disease due to hepatitis C virus	20 treatment,20 control	6 months	Significant improvement in liver function	No histological evidence; less detailed follow-up time points	[26]

*ALP* alkaline phosphatase, *BM-MSC* bone marrow-derived mesenchymal stem cell, *γ-GGT* glutamyl transpeptidase, *MELD* model for end-stage liver disease, *MSC* mesenchymal stem cell, *UC-MSC* umbilical cord-derived mesenchymal stem cell

Gholamrezanezhad et al. [27] have shown that there was no significant improvement in liver function after a 1-month period of follow-up because the homing ability of BM-MSCs into the liver occurred in just a limited number of infused cells. Peng et al. [28] also mentioned that the homing ability of MSCs is the main cause why autologous MSC transplantation did not achieve acceptable long-term effects on the prognosis of a patient. The lingering problem of cell-based therapies is whether the delivered cells home within the injured sites or not and how to increase their homing ability.

## Homing

Migration or homing within the injured tissues is influenced by multiple factors including the delivery route, the number of infused cells, culture conditions, and others. We review various factors that are related to the migration of MSCs.

### Administration routes of MSCs

The delivery route for MSCs seems to be crucial for therapeutic efficiency. Traditional administration of MSCs is mainly via intrahepatic injection, intrasplenic injection, and by intravenous infusion. Systemic delivery of cells may cause a large number of rapid losses of cells within the capillaries, especially in the lungs, which creates a short lifespan for remaining MSCs [29]. Furthermore, infusion of cells with heparin significantly decreases the number of entrapped AD-MSCs within the lungs and increases the number of cells which are accumulated in the liver [30]. The vascular patency may be an essential factor for MSCs flowing into the targeted tissue. Intrahepatic injection appeared to be the ideal way to administer stem cells, with less entrapment of cells in the circulation, and more MSCs differentiating into hepatocytes [31]. Furthermore, administration of the MSCs via the portal vein or hepatic artery shows homing efficacy less than 5% and 20–30%, respectively [32, 33]. The hepatic artery thus seems to be the best delivery route and shows better homing efficacy; however, the vascular patency should be checked before infusion.

### Optimizing cultivation conditions

During expansion, freshly isolated MSCs lose ligands or receptors which respond to migratory signals [34]. Migration is a passage-dependent process; with a higher number of passage there is a decrease in efficacy of homing. Also, high culture confluence impairs the migration of MSCs due to upregulation of tissue inhibitor of metalloproteinase (TIMP)-3 [35]. Moreover, hypoxia induces the expression of leptin which is associated with activation of both the STAT3/hypoxia-inducible factor-1α (HIF-1α)/VEGF and stromal cell-derived factor (SDF)-1/CXCR4 signaling pathways [36]. It is suggested that hypoxic preconditioning augments the recruitment of MSCs.

### Stimulating the target site to recruit MSC mobilization

In the acute phase of injury, inflammatory cytokines which were released from the damaged tissues recruit monocytes for tissues repair. Compared with unirradiated mice, more MSCs homed in mice that received total body irradiation [37], suggesting that infused MSCs are moved first to injured sites. However, in patients with a subchronic or chronic phase of the disease, some indispensable chemokines for homing may be minimal or absent; therefore external stimuli may provide a simple and available novel approach for homing.

Perry et al. [38] used degenerate electrical waveforms for patients with skin scars and showed that electrical stimulation significantly reduced scar scores and may guide cell migration. Furthermore, the physiological electrical field induced MSCs to graft to the anode in vitro, which had no influence on cell senescence and phenotype [39]. Meanwhile, pulsed focused ultrasound noninvasive local pressure waves deposit energy within the targeted tissues that change the level of local chemoattractants and enhance the efficacy of homing [40]. Mechanical stretching could also enhance engrafted MSC homing within injured tissues via hypoxia, vascularization, and proliferation [41]. In summary, external stimuli may be used to control or induce direct migration of MSCs.

### Genetically modified MSCs

Because of the presence of specific integration between ligand and receptor, one hypothesis is that changing the level of the receptor/ligand on MSCs may improve the efficiency of homing within the targeted tissues.

In the acute phase of injury, the damaged tissue releases numerous stromal cell-derived factors (SDF-1α), but their receptor (CXCR4) is at a low level on the cultured MSCs. MSCs with overexpressed CXCR4 have better migration potential toward SDF-1α and secrete more trophic factors, including HGF and VEGF which stimulate hepatocyte regeneration [42]. Ryu et al. [43] further explained that Akt, ERK, and p38 signal pathways are also related to the SDF-1/CXCR4 axis.

HGF is the most effective mitogen in hepatocyte regeneration and, during tissue injury, its biological effects rely on tyrosine kinase receptor and on cellular mesenchymal to epithelial transition factor (c-met) [44]. Genetic loss of c-met compromises the potential of hepatic oval cells, including their proliferation, migration, and differentiation [45]. Liu et al. [46] demonstrated that the HGF/c-met signaling pathway is crucial for MSC homing within the injured liver and that it facilitates the liver repair. Overexpressed receptor or ligands on the MSCs corresponds with the specific cytokines which are released from the injured organs and could induce homing directly within the targeted tissues.

MicroRNAs or noncoding RNAs target mRNA for degradation or inhibition and may determine the migration of MSCs. More than 60 different microRNAs in MSCs have been recently described and some of them are involved in migration, including let7, microRNA-10b, microRNA-27b, microRNA-335, and microRNA-886-3b [47]. Overexpressed microRNA-211 through the STAT3/microRNA-211/STAT5A signal pathway enhanced migration [48]. Upregulation of microRNA-221 and microRNA-26b enhanced MSC migration via the chemotactic response towards HGF through activation of PI3K/Akt signaling [49]. In addition, some other microRNAs suppressed migration of MSCs—microRNA-27b suppressed the directional migration of MSCs by targeting SDF-1α, and overexpression of microRNA-124 significantly inhibited the chemotactic migration towards HGF by downregulation of Wnt/β-catenin signaling [50]. It is suggested that microRNAs are involved in MSC potential, including their differentiation, paracrine function, proliferation, survival, and migration. Upregulation or downregulation of microRNAs in MSCs could regulate the migration.

## Conclusion

The present review demonstrates that stem cell therapy has a favorable therapeutic effect. Currently, the crucial factor that determines the benefit of MSCs is the homing efficacy. The disadvantages of MSC therapy in clinical trials include the risks of iatrogenic tumorigenesis, cellular embolism, and the optimum time for the infusion of cells. Moreover, its safety in clinical trials should be approved by institutional ethics committees. In conclusion, the results on MSCs which were used for the treatment of liver fibrosis are promising, but we need to know the underlying mechanism of their therapeutic effects.

## Abbreviations

AD-MSC: Adipose-derived mesenchymal stem cell
ALD: Alcoholic liver disease
BM-MSC: Bone marrow-derived mesenchymal stem cell
CB-MSC: Cord blood-derived mesenchymal stem cell
c-met: Cellular mesenchymal to epithelial transition factor
CXCR4: Chemokine receptor type 4
ECM: Extracellular matrix
EGF: Epidermal growth factor
ESC: Embryonic stem cell
HGF: Hepatocyte growth factor
HIF-1α: Hypoxia-inducible factor-1α
HPC: Hepatic progenitor cell
HSC: Hematopoietic stem cell
IGF-1: Insulin-like growth factor-1
iPSC: Induced pluripotent stem cell
MELD: Model for end-stage liver disease
MSC: Mesenchymal stem cell
NAFLD: Nonalcoholic fatty liver disease
NGF: Nerve growth factor
SDF: Stromal cell-derived factor
TGF: Transforming growth factor
TIMP: Tissue inhibitor of metalloproteinase
UC-MSC: Umbilical cord-derived mesenchymal stem cell
VEGF: Vascular endothelial growth factor

## References

[1] Lozano R, Naghavi M, Foreman K, Lim S, Shibuya K, Aboyans V, Abraham J, Adair T, Aggarwal R, Ahn SY, Alvarado M, Anderson HR, Anderson LM, Andrews KG, Atkinson C, Baddour LM, Barker-Collo S, Bartels DH, Bell ML, Benjamin EJ, Bennett D, Bhalla K, Bikbov B, Bin Abdulhak A, Birbeck G, Blyth F, Bolliger I, Boufous S, Bucello C, Burch M, Burney P, Carapetis J, Chen H, Chou D, Chugh SS, Coffeng LE, Colan SD, Colquhoun S, Colson KE, Condon J, Connor MD, Cooper LT, Corriere M, Cortinovis M, de Vaccaro KC, Couser W, Cowie BC, Criqui MH, Cross M, Dabhadkar KC, Dahodwala N, De Leo D, Degenhardt L, Delossantos A, Denenberg J, Des Jarlais DC, Dharmaratne SD, Dorsey ER, Driscoll T, Duber H, Ebel B, Erwin PJ, Espindola P, Ezzati M, Feigin V, Flaxman AD, Forouzanfar MH, Fowkes FG, Franklin R, Fransen M, Freeman MK, Gabriel SE, Gakidou E, Gaspari F, Gillum RF, Gonzalez-Medina D, Halasa YA, Haring D, Harrison JE, Havmoeller R, Hay RJ, Hoen B, Hotez PJ, Hoy D, Jacobsen KH, James SL, Jasrasaria R, Jayaraman S, Johns N, Karthikeyan G, Kassebaum N, Keren A, Khoo JP, Knowlton LM, Kobusingye O, Koranteng A, Krishnamurthi R, Lipnick M, Lipshultz SE, Ohno SL, Mabweijano J, MI MF, Mallinger L, March L, Marks GB, Marks R, Matsumori A, Matzopoulos R, Mayosi BM, McAnulty JH, McDermott MM, McGrath J, Mensah GA, Merriman TR, Michaud C, Miller M, Miller TR, Mock C, Mocumbi AO, Mokdad AA, Moran A, Mulholland K, Nair MN, Naldi L, Narayan KM, Nasseri K, Norman P, O'Donnell M, Omer SB, Ortblad K, Osborne R, Ozgediz D, Pahari B, Pandian JD, Rivero AP, Padilla RP, Perez-Ruiz F, Perico N, Phillips D, Pierce K, Pope CA, Porrini E, Pourmalek F, Raju M, Ranganathan D, Rehm JT, Rein DB, Remuzzi G, Rivara FP, Roberts T, De León FR, Rosenfeld LC, Rushton L, Sacco RL, Salomon JA, Sampson U, Sanman E, Schwebel DC, Segui-Gomez M, Shepard DS, Singh D, Singleton J, Sliwa K, Smith E, Steer A, Taylor JA, Thomas B, Tleyjeh IM, Towbin JA, Truelsen T, Undurraga EA, Venketasubramanian N, Vijayakumar L, Vos T, Wagner GR, Wang M, Wang W, Watt K, Weinstock MA, Weintraub R, Wilkinson JD, Woolf AD, Wulf S, Yeh PH, Yip P, Zabetian A, Zheng ZJ, Lopez AD, Murray CJ, AlMazroa MA, Memish ZA (2012). Global and regional mortality from 235 causes of death for 20 age groups in 1990 and 2010: a systematic analysis for the Global Burden of Disease Study 2010. Lancet.

[2] Freeman RB, Steffick DE, Guidinger MK, Farmer DG, Berg CL, Merion RM (2008). Liver and intestine transplantation in the United States, 1997–2006. Am J Transplant.

[3] Issa R, Zhou X, Constandinou CM, Fallowfield J, Millward-Sadler H, Gaca MD, Sands E, Suliman I, Trim N, Knorr A, Arthur MJ, Benyon RC, Iredale JP (2004). Spontaneous recovery from micronodular cirrhosis: evidence for incomplete resolution associated with matrix cross-linking. Gastroenterology..

[4] Sokal EM, Smets F, Bourgois A, Van Maldergem L, Buts JP, Reding R, Bernard Otte J, Evrard V, Latinne D, Vincent MF, Moser A, Soriano HE (2003). Hepatocyte transplantation in a 4-year-old girl with peroxisomal biogenesis disease: technique, safety, and metabolic follow-up. Transplantation..

[5] Ishii T, Yasuchika K, Machimoto T, Kamo N, Komori J, Konishi S, Suemori H, Nakatsuji N, Saito M, Kohno K, Uemoto S, Ikai I (2007). Transplantation of embryonic stem cell-derived endodermal cells into mice with induced lethal liver damage. Stem Cells..

[6] Miyajima A, Tanaka M, Itoh T (2014). Stem/progenitor cells in liver development, homeostasis, regeneration, and reprogramming. Cell Stem Cell..

[7] Schmelzer E, Zhang L, Bruce A, Wauthier E, Ludlow J, Yao HL, Moss N, Melhem A, McClelland R, Turner W, Kulik M, Sherwood S, Tallheden T, Cheng N, Furth ME, Reid LM (2007). Human hepatic stem cells from fetal and postnatal donors. J Exp Med..

[8] Li D, Cen J, Chen X, Conway EM, Ji Y, Hui L (2013). Hepatic loss of survivin impairs postnatal liver development and promotes expansion of hepatic progenitor cells in mice. Hepatology..

[9] Dubuquoy L, Louvet A, Lassailly G, Truant S, Boleslawski E, Artru F, Maggiotto F, Gantier E, Buob D, Leteurtre E, Cannesson A, Dharancy S, Moreno C, Pruvot FR, Bataller R, Mathurin P (2015). Progenitor cell expansion and impaired hepatocyte regeneration in explanted livers from alcoholic hepatitis. Gut..

[10] Nobili V, Carpino G, Alisi A, Franchitto A, Alpini G, De Vito R, Onori P, Alvaro D, Gaudio E (2012). Hepatic progenitor cells activation, fibrosis, and adipokines production in pediatric nonalcoholic fatty liver disease. Hepatology..

[11] Español-Suñer R, Carpentier R, Van Hul N, Legry V, Achouri Y, Cordi S, Jacquemin P, Lemaigre F, Leclercq IA (2012). Liver progenitor cells yield functional hepatocytes in response to chronic liver injury in mice. Gastroenterology..

[12] Libbrecht L, Desmet V, Van Damme B, Roskams T (2000). Deep intralobular extension of human hepatic “progenitor cells”correlates with parenchymal inflammation in chronic viral hepatitis: can “progenitor cells” migrate?. J Pathol..

[13] Xie G, Diehl AM (2013). Evidence for and against epithelial-to-mesenchymal transition in the liver. Am J Physiol Gastrointest Liver Physiol..

[14] Asahina K, Tsai SY, Li P, Ishii M, Maxson RE, Sucov HM, Tsukamoto H (2009). Mesenchymal origin of hepatic stellate cells, submesothelial cells, and perivascular mesenchymal cells during mouse liver development. Hepatology..

[15] Si-Tayeb K, Lemaigre FP, Duncan SA (2010). Organogenesis and development of the liver. Dev Cell..

[16] Lua I, James D, Wang J, Wang KS, Asahina K (2014). Mesodermal mesenchymal cells give rise to myofibroblasts, but not epithelial cells, in mouse liver injury. Hepatology..

[17] Jang YO, Kim MY, Cho MY, Baik SK, Cho YZ, Kwon SO (2014). Effect of bone marrow-derived mesenchymal stem cells on hepatic fibrosis in a thioacetamide-induced cirrhotic rat model. BMC Gastroenterol..

[18] Schubert T, Xhema D, Vériter S, Schubert M, Behets C, Delloye C, Gianello P, Dufrane D (2011). The enhanced performance of bone allografts using osteogenic-differentiated adipose-derived mesenchymal stem cells. Biomaterials..

[19] Baksh D, Yao R, Tuan RS (2007). Comparison of proliferative and multilineage differentiation potential of human mesenchymal stem cells derived from umbilical cord and bone marrow. Stem Cells..

[20] Mohammadi Gorji S, Karimpor Malekshah AA, Hashemi-Soteh MB, Rafiei A, Parivar K, Aghdami N (2012). Effect of mesenchymal stem cells on doxorubicin-induced fbrosis. Cell J..

[21] Eom YW, Shim KY, Baik SK (2015). Mesenchymal stem cell therapy for liver fibrosis. Korean J Intern Med..

[22] Zhang Z, Lin H, Shi M, Xu R, Fu J, Lv J, Chen L, Lv S, Li Y, Yu S, Geng H, Jin L, Lau GK, Wang FS (2012). Human umbilical cord mesenchymal stem cells improve liver function and ascites in decompensated liver cirrhosis patients. J Gastroenterol Hepatol..

[23] Wang L, Li J, Liu H, Li Y, Fu J, Sun Y, Xu R, Lin H, Wang S, Lv S, Chen L, Zou Z, Li B, Shi M, Zhang Z, Wang FS (2013). Pilot study of umbilical cord-derived mesenchymal stem cell transfusion in patients with primary biliary cirrhosis. J Gastroenterol Hepatol..

[24] El-Ansary M, Abdel-Aziz I, Mogawer S, Abdel-Hamid S, Hammam O, Teaema S, Wahdan M (2012). Phase II trial: undifferentiated versus differentiated autologous mesenchymal stem cells transplantation in Egyptian patients with HCV induced liver cirrhosis. Stem Cell Rev..

[25] Xu L, Gong Y, Wang B, Shi K, Hou Y, Wang L, Lin Z, Han Y, Lu L, Chen D, Lin X, Zeng Q, Feng W, Chen Y (2014). Randomized trial of autologous bone marrow mesenchymal stem cells transplantation for hepatitis B virus cirrhosis: regulation of Treg/Th17 cells. J Gastroenterol Hepatol..

[26] Salama H, Zekri AR, Medhat E, Al Alim SA, Ahmed OS, Bahnassy AA, Lotfy MM, Ahmed R, Musa S (2014). Peripheral vein infusion of autologous mesenchymal stem cells in Egyptian HCV positive patients with end stage liver disease. Stem Cell Res Ther..

[27] Gholamrezanezhad A, Mirpour S, Bagheri M, Mohamadnejad M, Alimoghaddam K, Abdolahzadeh L, Saghari M, Malekzadeh R (2011). In vivo tracking of 111In-oxine labeled mesenchymal stem cells following infusion in patients with advanced cirrhosis. Nucl Med Biol..

[28] Peng L, Xie DY, Lin BL, Liu J, Zhu HP, Xie C, Zheng YB, Gao ZL (2011). Autologous bone marrow mesenchymal stem cell transplantation in liver failure patients caused by hepatitis B: short-term and long-term outcomes. Hepatology..

[29] Zhang L, Li K, Liu X, Li D, Luo C, Fu B, Cui S, Zhu F, Zhao RC, Chen X (2013). Repeated systemic administration of human adipose-derived stem cells attenuates overt diabetic nephropathy in rats. Stem Cells Dev..

[30] Yukawa H, Watanabe M, Kaji N, Okamoto Y, Tokeshi M, Miyamoto Y, Noguchi H, Baba Y, Hayashi S (2012). Monitoring transplanted adipose tissue-derived stem cells combined with heparin in the liver by fluorescence imaging using quantum dots. Biomaterials..

[31] Chamberlain J, Yamagami T, Colletti E, Theise ND, Desai J, Frias A, Pixley J, Zanjani ED, Porada CD, Almeida-Porada G (2007). Efficient generation of human hepatocytes by the intrahepatic delivery of clonal human mesenchymal stem cells in fetal sheep. Hepatology..

[32] Puppi J, Strom SC, Hughes RD, Bansal S, Castell JV, Dagher I, Ellis EC, Nowak G, Ericzon BG, Fox IJ, Gómez-Lechón MJ, Guha C, Gupta S, Mitry RR, Ohashi K, Ott M, Reid LM, Roy-Chowdhury J, Sokal E, Weber A, Dhawan A (2012). Improving the techniques for human hepatocyte transplantation: report from a consensus meeting in London. Cell Transplant..

[33] Khan AA, Shaik MV, Parveen N, Rajendraprasad A, Aleem MA, Habeeb MA, Srinivas G, Raj TA, Tiwari SK, Kumaresan K, Venkateswarlu J, Pande G, Habibullah CM (2010). Human fetal liver-derived stem cell transplantation as supportive modality in the management of end-stage decompensated liver cirrhosis. Cell Transplant..

[34] Jin J (2009). Compared treatment of primary and repeated bone marrow mesenchymal stem cell transplantation on the acute myocardial infarction.

[35] De Becker A, Van Hummelen P, Bakkus M, Vande Broek I, De Wever J, De Waele M, Van Riet I (2007). Migration of culture-expanded human mesenchymal stem cells through bone marrow endothelium is regulated by matrix metalloproteinase-2 and tissue inhibitor of metalloproteinase-3. Haematologica..

[36] Hu X, Wu R, Jiang Z, Wang L, Chen P, Zhang L, Yang L, Wu Y, Chen H, Chen H, Xu Y, Zhou Y, Huang X, Webster KA, Yu H, Wang J (2014). Leptin signaling is required for augmented therapeutic properties of mesenchymal stem cells conferred by hypoxia preconditioning. Stem Cells..

[37] François S, Bensidhoum M, Mouiseddine M, Mazurier C, Allenet B, Semont A, Frick J, Saché A, Bouchet S, Thierry D, Gourmelon P, Gorin NC, Chapel A (2006). Local irradiation not only induces homing of human mesenchymal stem cells at exposed sites but promotes their widespread engraftment to multiple organs: a study of their quantitative distribution after irradiation damage. Stem Cells..

[38] Perry D, Colthurst J, Giddings P, McGrouther DA, Morris J, Bayat A (2010). Treatment of symptomatic abnormal skin scars with electrical stimulation. J Wound Care..

[39] Zhao Z, Watt C, Karystinou A, Roelofs AJ, McCaig CD, Gibson IR, De Bari C (2011). Directed migration of human bone marrow mesenchymal stem cells in a physiological direct current electric field. Eur Cell Mater..

[40] Burks SR, Ziadloo A, Kim SJ, Nguyen BA, Frank JA (2013). Noninvasive pulsed focused ultrasound allows spatiotemporal control of targeted homing for multiple stem cell types in murine skeletal muscle and the magnitude of cell homing can be increased through repeated applications. Stem Cells..

[41] Liang X, Huang X, Zhou Y, Jin R, Li Q (2016). Mechanical stretching promotes skin tissue regeneration via enhancing mesenchymal stem cell homing and transdifferentiation. Stem Cells Transl Med..

[42] Marquez-Curtis LA, Gul-Uludag H, Xu P, Chen J, Janowska-Wieczorek A (2013). CXCR4 transfection of cord blood mesenchymal stromal cells with the use of cationic liposome enhances their migration toward stromal cell-derived factor-1. Cytotherapy..

[43] Ryu CH, Park SA, Kim SM, Lim JY, Jeong CH, Jun JA, Oh JH, Park SH, Oh WI, Jeun SS (2010). Migration of human umbilical cord blood mesenchymal stem cells mediated by stromal cell-derived factor-1/CXCR4 axis via Akt, ERK, and p38 signal transduction pathways. Biochem Biophys Res Commun..

[44] Trusolino L, Bertotti A, Comoglio PM (2010). MET signalling: principles and functions in development, organ regeneration and cancer. Nat Rev Mol Cell Biol..

[45] Ishikawa T, Factor VM, Marquardt JU, Raggi C, Seo D, Kitade M, Conner EA, Thorgeirsson SS (2012). Hepatocyte growth factor (HGF)/c-met signaling is required for stem cell mediated liver regeneration. Hepatology..

[46] Liu J, Pan G, Liang T, Huang P (2014). HGF/c-Met signaling mediated mesenchymal stem cell-induced liver recovery in intestinal ischemia reperfusion model. Int J Med Sci..

[47] Clark EA, Kalomoiris S, Nolta JA, Fierro FA (2014). Concise review: microRNA function in multipotent mesenchymal stromal cells. Stem Cells..

[48] Hu X, Chen P, Wu Y, Wang K, Xu Y, Chen H, Zhang L, Wu R, Webster KA, Yu H, Zhu W, Wang J (2016). MiR-211/stat5a signaling modulates migration of mesenchymal stem cells to improve its therapeutic efficacy. Stem Cells..

[49] Zhu A, Kang N, He L, Li X, Xu X, Zhang H (2016). MiR-221 and miR-26b Regulate chemotactic migration of MSCs toward HGF through activation of Akt and FAK. J Cell Biochem..

[50] Yue Q, Zhang Y, Li X, He L, Hu Y, Wang X, Xu X, Shen Y, Zhang H (2016). MiR-124 suppresses the chemotactic migration of rat mesenchymal stem cells toward HGF by downregulating Wnt/β-catenin signaling. Eur J Cell Biol..

